# Practical Departments

**Published:** 1896-08-22

**Authors:** 


### PRACTICAL DEPARTMENTS.

### PASTEUR FILTERS.

The new filters made for the Darjiling Municipal Waterworks, on the system of filtration due to the late M. Pasteur and applied to drinking water by the present Chef de Service at the Pasteur Institute, Paris, have recently been on view at the makers, Messrs. J. Defries and Sons (Limited). The installation consists of 38 cells of tough cast iron, served with an acid-resisting composition, arranged in four rows. Each cell contains a number of Pasteur filter tubes fixed into solid elastic bushes, and is connected by wrought-iron pipes to cast-iron mains, which deliver into cast-iron collecting mains, all protected by the acid-resisting composition. The cells are fitted with gunmetal valves, enabling any one cell or group of cells to be cut out for cleaning or other reason. The inlet and outlet pipes are controlled by sluice valves in the ordinary way. Cleaning of the tubes and cells is effected by means of a circulating pump, fed from a small boiler fitted with Gresham's combination injector. This forces through the tubes of any cell or group of cells a solvent—usually a diluted acid—by which the dialytic deposit in the pores of the filter tubes is removed, and it is claimed that at the same time the whole of the filtering system is sterilised. This process only entails the passage of an inappreciable quantity of acid per gallon of filtered water during the day; and as the acid can be used again and again, it is accordingly both economical and free from objection. The whole of the parts of the installation are interchangeable, and the filter will be worked entirely by one man, who could, indeed, attend to one of much larger size. Its nominal output is 150,000 gallons per day; and it will be served by a gravitation supply under a net head of about 65 feet. The tubes are stated to wear for practically an indefinite period.

